# State of the Patients of the Bristol Dispensary, from the 1st of August to the 1st of September, 1801

**Published:** 1801-10

**Authors:** 


					[ 3?+ ]
Slate of the Patients of the Bristol Dispensary, from the \Jl qf
Augujl to the \Ji of September, 1801.
-Anafarca - - - - - 6
Afthma - - - - - - 4
Anorexia & Dyfpepfia - - 4
Abfcefius Mammarum - - 1
Afthenia ------ 3
Afcites - - - - - - 3
Angina Maligna - - - 2
Confumption - - *? - 7
Diarrhoea - - - t - 8
Dyfenteria ----- 8
Leucorrhcsa ----- 2
Menorrhcea ----- 2
Nervous ------ 1
Paralyfis ------ 1
Pleuritis ------ 2
Phthifis 3
Scorbutic - - - - 1
Synocha - - - - - 6
Typhus Fevers - - "53
Puerperal Fever - - - 1
Putrid Fevfer - - *

				

